# Pancreatic cancer as a sentinel for hereditary cancer predisposition

**DOI:** 10.1186/s12885-018-4573-5

**Published:** 2018-06-27

**Authors:** Erin L. Young, Bryony A. Thompson, Deborah W. Neklason, Matthew A. Firpo, Theresa Werner, Russell Bell, Justin Berger, Alison Fraser, Amanda Gammon, Cathryn Koptiuch, Wendy K. Kohlmann, Leigh Neumayer, David E. Goldgar, Sean J. Mulvihill, Lisa A. Cannon-Albright, Sean V. Tavtigian

**Affiliations:** 1 Department of Oncological Sciences, University of Utah School of Medicine, Salt Lake City, United States; 2 Huntsman Cancer Institute, University of Utah School of Medicine, Salt Lake City, United States; 3 Centre for Epidemiology and Biostatistics, School of Population and Global Health, University of Melbourne, Melbourne, Australia; 4 Division of Genetic Epidemiology, Department of Internal Medicine, University of Utah, Salt Lake City, United States; 5 Department of Surgery, University of Utah School of Medicine, Salt Lake City, United States; 6 Division of Oncology, Department of Medicine, University of Utah, Salt Lake City, United States; 7 Population Sciences, Huntsman Cancer Institute, University of Utah, Salt Lake City, United States; 8 Department of Surgery and Arizona Cancer Center, University of Arizona, Tucson, United States; 9 Department of Dermatology, University of Utah School of Medicine, Salt Lake City, United States; 10 George E. Wahlen Department of Veterans Affairs Medical Center, Salt Lake City, United States

**Keywords:** HBOC, Lynch syndrome, Colorectal cancer, Pancreatic cancer, Genetic testing

## Abstract

**Background:**

Genes associated with hereditary breast and ovarian cancer (HBOC) and colorectal cancer (CRC) predisposition have been shown to play a role in pancreatic cancer susceptibility. Growing evidence suggests that pancreatic cancer may be useful as a sentinel cancer to identify families that could benefit from HBOC or CRC surveillance, but to date pancreatic cancer is only considered an indication for genetic testing in the context of additional family history.

**Methods:**

Preliminary data generated at the Huntsman Cancer Hospital (HCH) included variants identified on a custom 34-gene panel or 59-gene panel including both known HBOC and CRC genes for respective sets of 66 and 147 pancreatic cancer cases, unselected for family history. Given the strength of preliminary data and corresponding literature, 61 sequential pancreatic cancer cases underwent a custom 14-gene clinical panel. Sequencing data from HCH pancreatic cancer cases, pancreatic cancer cases of the Cancer Genome Atlas (TCGA), and an unselected pancreatic cancer screen from the Mayo Clinic were combined in a meta-analysis to estimate the proportion of carriers with pathogenic and high probability of pathogenic variants of uncertain significance (HiP-VUS).

**Results:**

Approximately 8.6% of unselected pancreatic cancer cases at the HCH carried a variant with potential HBOC or CRC screening recommendations. A meta-analysis of unselected pancreatic cancer cases revealed that approximately 11.5% carry a pathogenic variant or HiP-VUS.

**Conclusion:**

With the inclusion of both HBOC and CRC susceptibility genes in a panel test, unselected pancreatic cancer cases act as a useful sentinel cancer to identify asymptomatic at-risk relatives who could benefit from relevant HBOC and CRC surveillance measures.

**Electronic supplementary material:**

The online version of this article (10.1186/s12885-018-4573-5) contains supplementary material, which is available to authorized users.

## Background

Over the last few years, massively parallel sequencing converged with targeted capture using array synthesized baits to enable panel testing of most known cancer susceptibility genes [1–4]. These panel tests have since replaced Sanger sequencing of limited sets of syndromic genes, thereby revolutionizing the genetic testing landscape for Hereditary Breast and Ovarian cancer (HBOC) and Colorectal cancer (CRC) predisposition. A great benefit for these predictive genetic tests is to identify carriers of pathogenic medically-actionable variants in asymptomatic individuals, notably the at-risk relatives of the sentinel cancer patient.

Methods for prevention or early detection of pancreatic cancer have limited utility, [5, 6] so utilizing germline predisposition testing to identify individuals with modest to moderate increases in pancreatic cancer susceptibility is also limited in utility. Current guidelines recommend genetic testing only in pancreatic cancer patients with additional family history matching the patterns indicative of hereditary breast and ovarian cancer, colorectal cancer predisposition such as FAP or Lynch syndrome, or melanoma [7–11]. However, studies have found that these criteria will miss 50% of pancreatic cancer patients who harbor actionable pathogenic variants [12, 13]. Therefore, pancreatic cancer may be a useful sentinel cancer for identification of carriers of pathogenic variants in HBOC and CRC susceptibility genes, whose relatives can benefit from surveillance, medical, and surgical strategies for prevention, risk reduction, or early detection [7, 14–22].

To estimate the percentage of pancreatic cancer cases that carry variants with potential medical management impact for at-risk relatives, we applied panel testing to 274 pancreatic cancer patients ascertained at the Huntsman Cancer Hospital (HCH) in Salt Lake City, UT, unselected for family cancer history. To demonstrate generalizability of the results in pancreatic cancer cases, we performed a meta-analysis including published panel tests of unselected pancreatic cancer cases.

## Methods

### Subjects and ethics statement

This study was approved by the Institutional Review Board of the University of Utah. All participants gave written consent, which included DNA sampling for molecular studies and access to medical records.

An initial set of pancreatic cancer cases (*n* = 66) were selected on the minimal requirements of personal history of cancer and having at least two grandparents in the genealogy data represented in the Utah Population Database (UPDB). These patients were screened with a 34-gene custom research panel. Individual family members were then linked to statewide cancer, demographic, and medical information [23]. Ages at diagnosis and family cancer history were obtained from the UPDB after sequencing and variant evaluation. Additional subjects were selected on the basis of being newly diagnosed pancreatic cancer cases ascertained at the HCH from July 2014 to April 2017 (*n* = 224). The pancreatic cancer cases ascertained during the interval July 2014–November 2015 (*n* = 151) were screened with a 59-gene custom research panel, and the cases ascertained during the interval December 2015–April 2017 (*n* = 73) were screened with a 14-gene custom clinical panel.

### Next-generation sequencing library preparation and custom targeted capture

For research panel testing, blood-derived genomic DNA (100 ng) was sheared using a Covaris S2 instrument (Covaris, Woburn, MA, United States). Genomic libraries were prepared using the Ovation Ultralow Library System (NUGEN # 0329) according to the manufacturer’s instructions. Library enrichment for a 34 or 59-gene custom panel was done with the Roche SeqCap EZ Choice Library (cat# 06266339001) and the SeqCap EZ Reagent Kit Plus v2 (NimbleGen #06–953–247-001) using the manufacturer’s protocol. Individual libraries were combined into pools of 6–12 prior to hybridization, and then super-pooled for up to 96 samples per sequencing lane. Captured libraries were sequenced on an Illumina HiSeq2000 channel using the HiSeq 101 Cycle Paired-End sequencing protocol.

On the strength of preliminary data from this study, HCH began systematically offering clinical panel predisposition testing beginning December 2015, without regard to family history. From December 2015 to April 2017, clinical testing was offered to 73 sequential pancreatic cancer cases. Sixty-one pancreatic cancer patients accepted clinical testing with the 14-gene custom panel that was conducted by Invitae. The 12 individuals that declined were in poor health and/or did not see value in undergoing a genetic test. A complete list of genes captured is included in Additional file 1: Table S1.

Sequences from the Utah cohort with ≥100X mean coverage and 154 pancreatic cancer cases from the Cancer Genome Atlas (TCGA) [24] were analyzed using the USeq (useq.sourceforge.net) in-house pipeline, according to the Genome Analysis Toolkit (GATK v.3.3–0) best practices recommendations [25]. Variants with a mapping quality score less than 20 were excluded. ANNOVAR was used for variant functional annotation followed by conversion to Human Genome Variation Society (HGVS) nomenclature using Mutalyzer [26, 27].

### Sequence variant evaluation

Truncating variants not present in the final exon of a gene were considered pathogenic. The following filters were used to exclude variants from further analysis: minor allele frequency ≥ 0.1% in one or more populations from the Exome Aggregation Consortium (ExAC) database; [28] synonymous/intronic variants with no predicted effect on splicing via MaxEntScan; [29] variants reported as probable-non-pathogenic/non-pathogenic by more than one source with no conflicting reports in ClinVar (www.ncbi.nlm.nih.gov/clinvar).

**Fig. 1:**
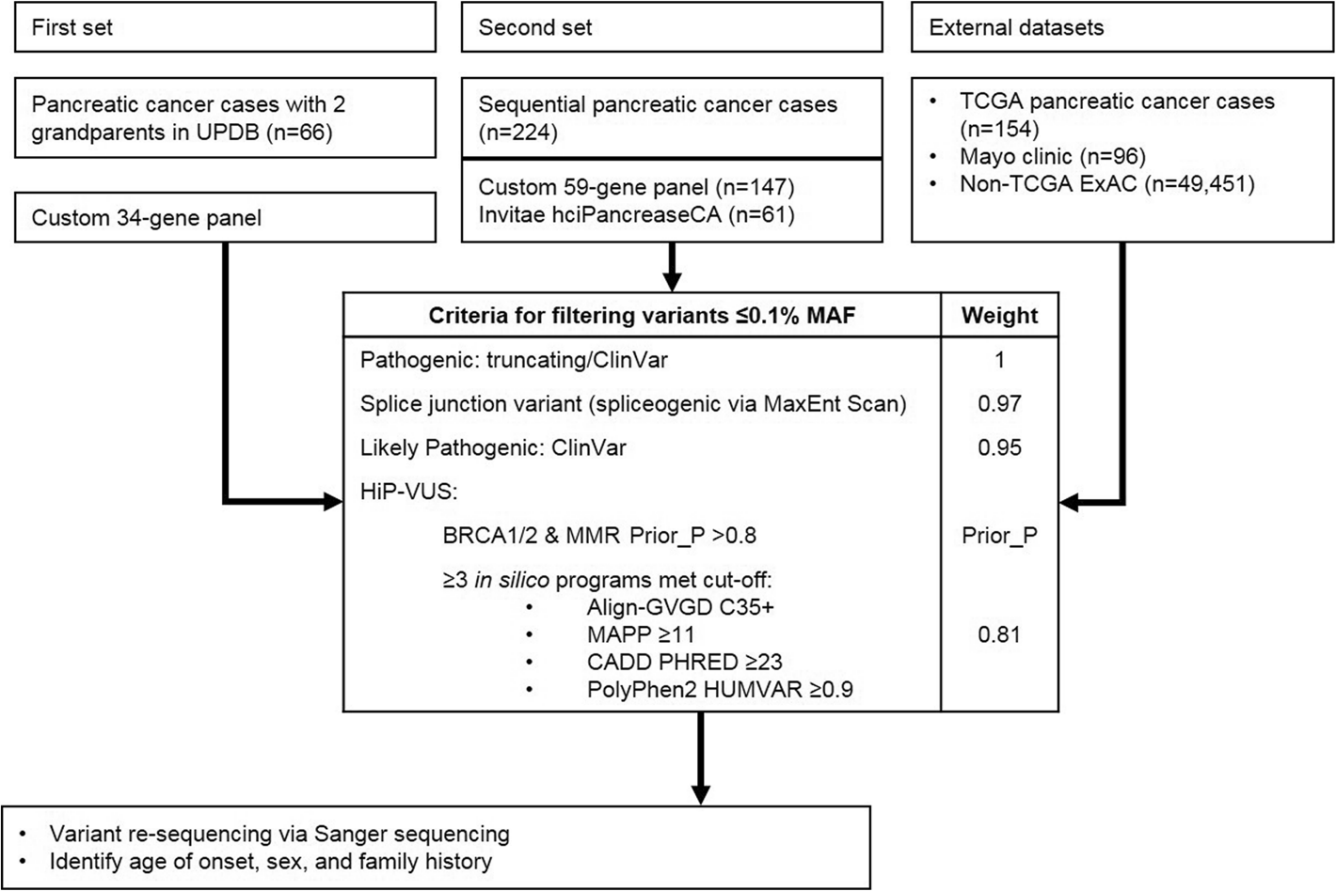
Flow chart of methods. Includes the weighting assigned to filtered sequence variants. In the second set of unselected pancreatic cancer cases 16 cases failed testing with the custom 59-gene panel and/or declined testing with the Invitae hciPancreasCA panel. MAF = minor allele frequency; HiP-VUS = high probability of pathogenicity variant of uncertain significance; MMR – mismatch repair; Prior_P = sequence analysis-based prior probability of pathogenicity

Variants of uncertain significance (VUS) were included if in silico predictions were suggestive of being relatively high probabilities of pathogenicity VUS (HiP-VUS). HiP-VUS had estimated prior probabilities of pathogenicity > 0.8 based on calibrated in silico predictions from publicly available databases for the mismatch repair (MMR) genes (hci-lovd.hci.utah.edu), or *BRCA1*/2 (http://priors.hci.utah.edu/PRIORS/). HiP-VUS of this type were weighted according to their sequence analysis-based prior probability of pathogenicity (Prior_P) score. The VUS from the remaining genes were denoted HiP-VUS and included if at least three of the four missense analysis programs Align-GVGD, MAPP, Polyphen-2, and CADD predicted a severe score [30–34]. This filter corresponds with an OR = 3.27 when comparing early-onset breast cancer cases with matched controls [33]. Based on the likelihood ratios identified for *BRCA1*/2, [35] this grouping was assigned a weight of 0.81. Canonical splice acceptor/donor variants predicted to impact splicing were given the weight of 0.97 if the effect of the variant had not been demonstrated experimentally [36]. Pathogenic variants and HiP-VUS detected by the 34−/59-gene panels were confirmed via Sanger sequencing. VUS reported by the Mayo Clinic [37] plus the non-TCGA ExAC (excluding the Finnish and undescribed populations), were graded with the same weights and severity to generate a bioinformatically equivalent set of HiP-VUS. An overview of the datasets and methods used for evaluation is shown (Fig. 1).

### Statistical analysis

STATA V.13.1 (StataCorp, College Station, Texas, USA) was used to conduct meta-analyses, and calculate carrier percentages and 95% confidence intervals. The meta-analyses to compare the carrier frequencies between different pancreatic cancer cohorts were conducted using Metaprop under a random effects model, and Freeman-Tukey transformation to stabilize the variances over the studies [38]. The weighted proportions of variant carriers in the unselected pancreatic cancer cases were compared to the corresponding proportions in the non-TCGA ExAC population to estimate Standardized Incidence Ratios (SIR) [39]. Tests of significance and confidence intervals were estimated based on a Poisson distribution [40]. For the meta-analysis and SIR calculation, the genes were split into subgroups of high- and moderate-risk cancer susceptibility genes. High- and moderate-risk were defined as genes with a cumulative risk at age 80 > 32% or between 19 and 32%, respectively, for the cancer with which they are most closely associated [2]. The R package ggplot2 was used to plot the meta-analyses and SIRs [41].

## Results

### Identification of pathogenic variants and HiP-VUS in pancreatic cancer patients, unselected for family history

In an initial set of 66 pancreatic cancer cases unselected for family history of cancer, 4 pathogenic variants were identified in *BRCA2*, *MSH6*, *PALB2*, and *STK11*. After filtering VUS, 2 HiP-VUS in *ATM* remained (Table 1). After weighting, 8.5% of these pancreatic cancer cases carried a variant with potential medical management impact for relatives.

**Table 1:** Pathogenic variants and high probability of pathogenicity variants of uncertain significance (HiP-VUS) identified from a custom 34- or 59-gene panel and a clinical 14-gene panel in pancreatic cancer cases, unselected for family history

Gene	HGVS Notation	Sex: Age of Onset	Carrier Weight
Custom 34-gene panel (*n* = 66)			
ATM	c.7327C > G p.(R2443G)	M: 60s	0.81
ATM	c.8734A > G p.(R2912G)	F: 60s	0.81
BRCA2	c.3873del p.(Q1291Hfs*2)	M: 50s	1
MSH6	c.3261dup p.(F1088Lfs*5)	F: 50s	1
PALB2	c.1240C > T p.(R414*)	F: 60s	1
STK11	c.738C > A p.(Y246*)	M: 40s	1
	Carrier Frequency: 5.62/66 = 8.52% (3.87–17.7%)		
Custom 59-gene panel (*n* = 147)			
ATM	c.1564_1565del p.(E522Ifs*43)	M: 50s	1
ATM	c.8734A > G p.(R2912G)	M: 70s	0.81
BRCA1	c.68_69del p.(E23Vfs*17)	M: 70s	1
BRCA2	c.3974_3975insTGCT p.(T1325Cfs*4)	M: 70s	1
BRCA2	c.8447G > A p.(G2816D)	F: 60s	0.81
CHEK2	c.1159A > G p.(T387A)	M: 80s	0.81
CHEK2	c.1427C > T p.(T476 M)	F: 50s	0.99^a^
MRE11A	c.923dupT p.(M309Hfs*8)	M: 50s	1
MRE11A	c.1516G > T p.(E506*)	F: 60s	1
MSH6	c.3851C > T p.(T1284 M)	F: 60s	0.94
PALB2	c.2167_2168del p.(M723Vfs*21)	M: 60s^c^	1
RAD50	c.3641G > A p.(R1214H)	F: 50s	-^a^
TP53	c.847C > T p.(R283C)	M: 60s	0.81
Clinical 14-gene panel (*n* = 61)			
ATM	c.1402_1406delAAGAG p.(K468Vfs*17)	F:40s	1
ATM	c.2426C > A p.(S809*)	F:80s^d^	1
ATM	c.3993 + 1G > A (splice donor)	M:70s^e^	-^b^
BRCA2	c.6275_6276delTT p.(L2092Pfs*7)	M:70s	1^b^
CDKN2A	c.301G > T p.(G101 W)	F:60s^f^	1
CHEK2	c.349A > G p.(R117G)	F:70s	0.95
MSH6	c.1444C > T p.(R482*)	F:70s^g^	1
TP53	c.1015G > A p.(E339K)	F:70s	0.81
	Carrier Frequency: 17.89/208 = 8.60% (5.50–13.20%)		

*HGVS* Human Genome Variation Society

^a,b^The same individual carried both variants, so carrier weight was combined and only counted once. Additional cancers: ^c^CRC in 40s; ^d^lung in 80s and CRC in 60s; ^e^prostate (Gleason 7) in 70s; ^f^melanoma in 40s and cervical in 30s; ^g^breast in 50s, endometrial in 50s, and urethral in 70s

Two series of cases were used for internal replication. From a set of 147 pancreatic cancer cases undergoing an in-house custom 59-gene panel, 6 pathogenic variants were identified in *ATM*, *BRCA1*, *BRCA2*, *MRE11A*, and *PALB2*, and 6 HiP-VUS in *ATM*, *BRCA2*, *CHEK2*, *MSH6*, and *TP53*. In addition to in silico predictions, *CHEK2* p.(T476 M) was found to be damaging in a functional assay for *CHEK2* variants, and was thus weighted more strongly towards being pathogenic [42]. Subsequently, an additional set of pancreatic cancer cases (*n* = 61) were tested with the 14-gene clinical panel, which identified 6 pathogenic variants and 2 HiP-VUS in 7 patients (Table 1). All pathogenic variants and HiP-VUS were identified in genes included in the original 34-gene panel. After weighting carriers, 8.6% of the pancreatic cancer cases from the latter two sets, unselected for family history, carried a variant with potential medical impact. A full list of rare variants with corresponding *in silico* information is available in Additional file 1: Table S2.

### Post-variant evaluation of genetic testing eligibility

In order to estimate the proportion of pancreatic cancer cases with pathogenic variants or HiP-VUS that would have qualified for genetic testing, the family histories were compared to NCCN guidelines [43]. Pedigree data regarding familial cancer were available from UPDB for the carriers of pathogenic variants and HiP-VUS in 6 of the initial set of 66 pancreatic cancer patients (Additional file 2: Figure S1). Self-reported family history information was also available for the final set of 61 patients that underwent the Invitae clinical panel, including the 7 carriers of pathogenic variants or HiP-VUS. Among the 13 identified carriers, the STK11 carrier had a clinical diagnosis of Peutz-Jegher syndrome with multiple affected relatives, and 9 additional patients met criteria for HBOC or Lynch syndrome genetic testing. Thus, more than 23% of these carriers would not have qualified for testing under current guidelines.

Once a pathogenic variant is identified, cascade testing for that variant can occur on biological relatives to identify individuals who would benefit from HBOC or CRC preventive measures. For three of the five pancreatic cancer cases with positive results with the clinical panel, 17 biological relatives have undergone cascade genetic testing thus far, 10 of whom have tested positive for the family pathogenic variant. Of note, 21 (29%) pancreatic cancer cases who underwent the clinical panel passed away since the beginning of the study, 17 of whom had undergone testing. This suggests that having a family member(s) present during pre-test counseling and delegated to receive results may be beneficial in the context of utilization of pancreatic cancer as a sentinel for HBOC or Lynch syndrome.

### Meta-analysis of carrier proportions across studies

**Fig. 2:**
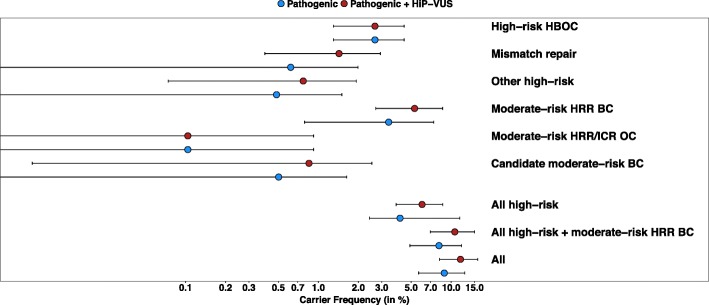
Proportion of carriers of pathogenic variants and high probability of pathogenicity variants of uncertain significance (HiP-VUS) in unselected pancreatic cancer. Results based on a meta-analysis of the unselected pancreatic cancer cases from the Huntsman Cancer Hospital (HCH), the Mayo Clinic, and the pancreatic cancer cases from The Cancer Genome Atlas (TCGA). Carrier frequency point estimates and 95% confidence intervals for groups of genes are presented on a log-scale. A list of genes contained within each analysis group is provided in Additional file 1: Table S1. The breakdown of results by study is described in Additional file 1: Table S3. HBOC = Hereditary Breast and Ovarian Cancer; HRR = Homologous Recombination and Repair; ICR = Interstrand Crosslink Repair; OC = Ovarian Cancer; BC = Breast Cancer

The HCH sets of pancreatic cancer cases were combined with a published study of unselected pancreatic cancer cases from the Mayo Clinic (*n* = 96), [37] plus the pancreatic cancer cases from TCGA (*n* = 154), in a meta-analysis (Fig. 2; Additional file 1: Table S3; Additional file 3: Table S4). Among unselected pancreatic cancer cases, 3.9% (*p* = 2.1 × 10^− 13^) carried a clearly pathogenic variant in a high-risk cancer susceptibility gene which includes pancreatic cancer in its tumor spectrum [8]. Weighed inclusion of HiP-VUS increased the proportion to 5.1% (*p* = 6.8 × 10^− 18^). For the moderate-risk homologous recombination repair (HRR) breast cancer genes *ATM*, *BARD1*, *CHEK2*, and *NBN*, [2, 44] 3.3% (*p* = 7.2 × 10^− 04^) of unselected pancreatic cancer cases carried a clearly pathogenic variant, and weighted inclusion of HiP-VUS increased the proportion to 5.1% (*p* = 2.3 × 10^− 08^). Including all the high-risk genes and the moderate-risk HRR breast cancer genes, 7.9% (*p* = 1.4 × 10^− 12^) of unselected pancreatic cancer cases carry a clearly pathogenic variant, and 10.4% (*p* = 9.1 × 10^− 17^) carry either a clearly pathogenic variant or weighted HiP-VUS with elevated probability of pathogenicity that could enable the at-risk relatives to qualify for preventive HBOC or CRC measures.

**Fig. 3:**
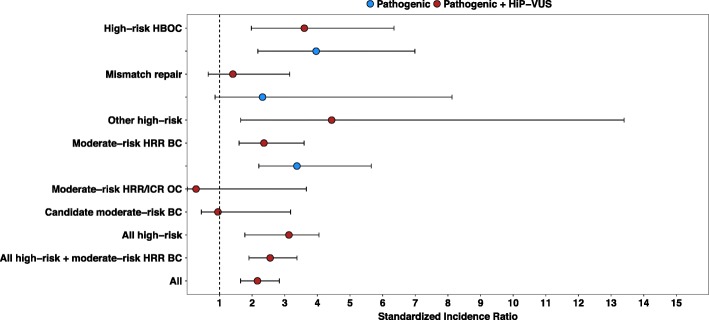
Standardized incidence ratios for cancer susceptibility gene groups in unselected pancreatic cancer cases. The carrier frequencies from the meta-analysis of the cases and the Exome Aggregation Consortium excluding the Cancer Genome Atlas (non-TCGA ExAC) are detailed in Additional file 3: Table S5. A list of genes contained within each analysis group is provided in Additional file 1: Table S1. Error bars represent 95% confidence intervals. HBOC = Hereditary Breast and Ovarian Cancer; HRR = Homologous Recombination and Repair; ICR = Interstrand Crosslink Repair; OC = Ovarian Cancer; BC = Breast Cancer

Further, the gene burdens observed in the Utah, Mayo, and TCGA were compared to the non-TCGA ExAC (*n* = 49,451, excluding the Finnish and other subpopulations) as a population sample to determine SIR for subgroups of genes (Fig. 3, Additional file 3: Table S5). As a group, the high-risk susceptibility genes had a SIR = 2.6 (*p* = 1.6 × 10^− 05^). The moderate-risk HRR genes had a slightly lower SIR = 2.3 (*p* = 2.0 × 10^− 05^).

## Discussion

From the 16 pathogenic and 11 HiP-VUS carriers identified through systematic panel testing of pancreatic cancer cases unselected for family history, we estimate that 2.7% (95% CI: 1.4–4.4) carry a pathogenic allele of a high-risk HBOC gene (*BRCA1*, *BRCA2*, or *PALB2*) and 1.3% (95% CI: 0.3–2.8) of probands carry a pathogenic allele of a Lynch Syndrome (LS)-associated MMR gene (*MLH1*, *MSH2*, *PMS2*, or *MSH6*). Adding other high-risk genes such as *TP53*, *CDKN2A*, and *STK11* results in 5.1% (95% CI: 3.3–7.2) of pancreatic cancer cases with a sequence variant that would alter medical management of healthy at-risk relatives: i.e. MRI in addition to mammography or early colonoscopy (Fig. 2) [7, 9, 45–47]. An additional 5.1% (95% CI: 2.4–8.5) are estimated to carry a pathogenic allele of a moderate-risk breast cancer susceptibility gene (i.e., *ATM*, *BARD1*, *CHEK2,* or *NBN*) bringing the total proportion of estimated carriers to 10.4% (95% CI: 6.5–14.9). Here we note that *ATM* and *CHEK2* have recently been added to NCCN’s list of genes with associated medical action for breast cancer [7]. Focusing on individual genes, the top four genes with potential medical impact for at-risk relatives, based on weighted counts, were *ATM* (identified in 7 cases)*, BRCA2* (4 cases)*, CHEK2* (3 cases)*,* and *MSH6* (3 cases).

The precedent for testing all pancreatic cancer patients comes from what has been accepted and learned from universal testing of all CRCs for LS. Universal LS testing, with immunohistochemical (IHC) or microsatellite instability (MSI)-based pre-screen of tumors followed by germline testing for indicated individuals, is recommended for newly diagnosed CRC cases [45, 48]. This strategy may soon be overtaken by germline DNA panel testing for LS due to 1) rapid decline of panel testing cost, 2) superiority of specificity and sensitivity, and 3) evidence that pre-screening delays genetic testing, which results in a subsequent ~ 50% loss in follow up by patients [49–56]. Indeed, a health economics analysis recently published by Erten et al. [56] concluded that universal testing of CRC patients for LS based on sequencing alone will become more cost effective than the two-step test when the cost of MMR gene sequencing drops to or below $609 USD, echoing a similar finding by Gould-Suarez et al. [56, 57]. Based on our results, universal testing of pancreatic cancer patients using a panel test would identify pathogenic variants or HiP-VUS in MMR genes at a frequency similar to what is detected with universal screening of CRC patients, 1.3 and 1.2% respectively [56].

In a recent study, 11.8% of unselected patients with metastatic prostate cancer were found to carry pathogenic variants in DNA-repair genes [58]. Pritchard et al. suggest that this proportion of metastatic prostate cancer cases is high enough to utilize metastatic prostate cancer as a sentinel for cancer predisposition testing. The 11.8% proportion observed in metastatic prostate cancer is similar to the 10.4% observed in this meta-analysis of pancreatic cancer cases. For these patients, universal panel testing offers critical time and convenience advantages over step-wise testing strategies, resulting in decreased loss to follow up or mortality and correspondingly increased benefit to at-risk relatives.

Lastly, there are an increasing number of options for targeted treatments based on germline mutations. PARP inhibitors show promise in pancreatic cancer, include olaparib and rucaparib (which are FDA approved in ovarian cancer), as well as veliparib (or ABT-888) which is in clinical trials [59]. Most patients with pathogenic germline *BRCA1*, *BRCA2*, or *PALB2* variants would be expected to respond. Solid tumors with MMR deficiency often respond to immunotherapy [60]. The US Food and Drug Administration (FDA) has granted accelerated approval to pembrolizumab (Keytruda) for pediatric and adult patients with microsatellite unstable cancers, a hallmark molecular feature of Lynch syndrome related cancers [59]. The availability of targeted treatments increases the utility of testing for pancreatic cancer patients themselves, in addition to the prevention and screening benefits for relatives.

## Conclusions

Our study adds to the increasing body of evidence that pancreatic cancer is an indicator of hereditary cancer predisposition. Identifying cases who carry mutations in genes associated with clinical recommendations will allow relatives to benefit from screening and prevention strategies for the range of cancer risks conferred. Increasingly, the finding of a germline mutation in pancreatic cancer patients may also impact their treatment. The benefits of genetic testing of all pancreatic cancer cases mirrors other cancers for which routine evaluation has become standard of care. Significant morbidity and poor prognosis may make this a uniquely challenging population to offer genetic counseling and testing. Research on the inherited basis of pancreatic cancer should be paired with psychosocial and behavioral studies to determine how best to incorporate genetic testing into the care of these patients and to ensure that findings are optimally used to benefit families.

## Additional files

Additional file 1: **Table S1.** contains the list of the genes used in the study, as well as indicators for which pathway they belong to in subsequent analyses. **Table S2.** is the list of rare variants with corresponding in silico scores. **Table S3.** contains the total counts that were used to create Fig. 2. (XLSX 73 kb)

Additional file 2: **Figure S1.** The pancreatic cancer cases with Utah Population Database (UPDB) genealogies with cancer information. (JPG 847 kb)

Additional file 3: **Table S4.** Is a list of reports/genes used for meta-analysis. **Table S5.** is the values that were used to create Fig. 3. (DOCX 34 kb)

## Abbreviations

CRC: colorectal cancer
ExAC: Exome Aggregation Consortium
FDA: US Food and Drug Administration
GATK: Genome Analysis Toolkit
HBOC: hereditary breast and ovarian cancer
HCH: Huntsman Cancer Hospital
HGVS: Human Genome Variation Society
HiP-VUS: high probabilities of pathogenicity variants of uncertain significance
IHC: immunohistochemical
LS: Lynch Syndrome
MMR: mismatch repair
MSI: microsatellite instability
SIR: Standardized Incidence Ratios
TCGA: the Cancer Genome Atlas
UPDB: Utah Population Database
VUS: Variants of uncertain significance
