# Supplementary figures and images for: Pancreatic cancer as a sentinel for hereditary cancer predisposition
Source: BMC Cancer. 2018 Jun 27;18:697. doi: 10.1186/s12885-018-4573-5 (PMC6020441; doi:10.1186/s12885-018-4573-5)

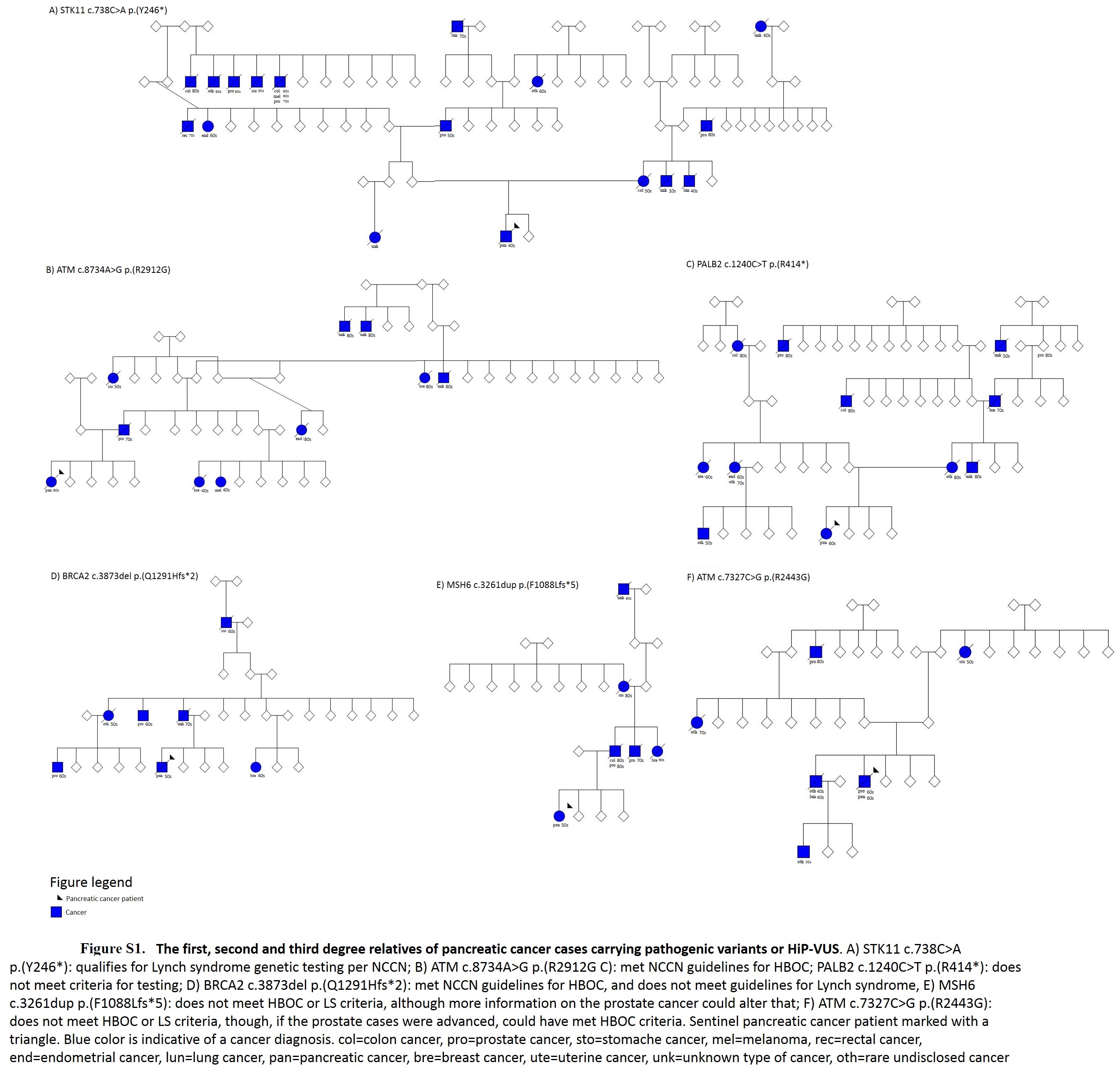

Supplement: Supplementary file 2 — Figure S1. The pancreatic cancer cases with Utah Population Database (UPDB) genealogies with cancer information. (JPG 847 kb) [file 12885_2018_4573_MOESM2_ESM.jpg]
