# Supplementary material for: Pancreatic cancer as a sentinel for hereditary cancer predisposition
Source: BMC Cancer. 2018 Jun 27;18:697. doi: 10.1186/s12885-018-4573-5 (PMC6020441; doi:10.1186/s12885-018-4573-5)
Supplement: Supplementary file 3 — Table S4. Is a list of reports/genes used for meta-analysis. Table S5. is the values that were used to create Fig. 3. (DOCX 34 kb) [file 12885_2018_4573_MOESM3_ESM.docx]

| Table S4. List of studies used for meta-analysis and standardized incidence ratio (SIR) calculations | | | |
| --- | --- | --- | --- |
| Study | Sample Size | Selection Criteria | Genes Reported |
| HCH PC 1 | 66 | Unselected | “All Genes” Column in Table S1 |
| HCH PC 2 | 208 | Unselected | “All Genes” Column in Table S1 |
| TCGA PC Cases^1^ | 154 |  | “All Genes” Column in Table S1 |
| Hu 2015^2^ | 96 | Unselected | BRCA1, BRCA2, PALB2, ATM, BARD1, BRIP1, RAD51C, RAD51D, CHEK2, MRE11A, NBN, RAD50, MLH1, MSH2, MSH6, PMS2, CDH1,TP53, PTEN, STK11, FANCM |
| Non-TCGA ExAC^1^ | 49,451 | Excluding FIN and OTH subgroups | “All Genes” Column in Table S1 |
| HCH = Huntsman Cancer Hospital, SLC, UT; PC = Pancreatic Cancer; TCGA = The Cancer Genome Atlas | | | |

| Table S5: Table of standardized incidence ratios for cancer susceptibility gene groups in unselected pancreatic cancer cases | | | | | | |
| --- | --- | --- | --- | --- | --- | --- |
| Gene Category | No. of carriers  (weighted) | Case carrier frequency | Non-TCGA ExAC (weighted) | Non-TCGA ExAC carrier frequency | SIR | 95%-CI |
| High-risk HBOC | 15 (14.8) | 0.027 | 369 (358.7) | 7.42e-3 | 3.68 | [2.06; 6.20] |
| Pathogenic^a^ | 14 (14) | 0.026 | 328 (325.4) | 6.74e-3 | 3.79 | [2.17; 6.65] |
| Mismatch repair | 8 (7.69) | 0.013 | 526 (484.6) | 0.010 | 1.27 | [0.59; 2.84] |
| Pathogenic^a^ | 4 (4) | 5.45e-3 | 130 (128.4) | 2.67e-3 | 2.04 | [0.78; 7.32] |
| Other high-risk^b^ | 5 (4.62) | 6.95e-3 | 88 (79.7) | 1.73e-3 | 4.01 | [1.49; 12.1] |
| Moderate-risk HRR BC | 29 (27.3) | 0.051 | 968 (873.3) | 0.022 | 2.25 | [1.51; 3.34] |
| Pathogenic^a^ | 21 (20.8) | 0.033 | 538 (531.3) | 0.011 | 2.90 | [2.12; 5.29] |
| Moderate-risk HRR/ICR OC^c^ | 1 (1) | 7.5e-4 | 197 (181.2) | 3.70e-3 | 0.20 | [0.01; 3.25] |
| Candidate moderate-risk BC^d^ | 7 (6.43) | 9.23e-3 | 485 (430.4) | 8.93e-3 | 1.03 | [0.53; 3.16] |
| All high-risk | 28 (27.1) | 0.051 | 983 (923.0) | 0.019 | 2.62 | [1.76; 3.88] |
| All high-risk &  moderate-risk HRR BC | 56 (53.4) | 0.104 | 1951 (1796.3) | 0.042 | 2.48 | [1.81; 3.17] |
| All | 65 (61.1) | 0.115 | 2633 (2407.9) | 0.054 | 2.11 | [1.64; 2.75] |

^a^Includes truncating, splice junction, pathogenic, and likely pathogenic variants reported in all PC studies in Table S4. ^b^*APC*, *BMPR1A*, *SMAD4*, ^c^*RAD51D*, and ^d^*NF1* were excluded from the analysis, because they were not screened in all studies. HBOC = Hereditary Breast and Ovarian Cancer; HRR = Homologous Recombination and Repair; ICR = Interstrand Crosslink Repair; OC = Ovarian Cancer; BC = Breast Cancer; 95%-CI = 95% Confidence Interval.

References:

1. Lek M, Karczewski K, Minikel E, et al. Analysis of protein-coding genetic variation in 60,706 humans. bioRxiv 2015:1–26. Available at: http://biorxiv.org/content/early/2015/10/30/030338.abstract.

2. Hu C, Hart SN, Bamlet WR, et al. Prevalence of Pathogenic Mutations in Cancer Predisposition Genes among Pancreatic Cancer Patients. Cancer Epidemiol. Biomarkers Prev. 2016;25:207–11. Available at: http://cebp.aacrjournals.org/cgi/doi/10.1158/1055-9965.EPI-15-0455.

3. Catts ZA-K, Baig MK, Milewski B, et al. Statewide Retrospective Review of Familial Pancreatic Cancer in Delaware, and Frequency of Genetic Mutations in Pancreatic Cancer Kindreds. Ann. Surg. Oncol. 2016;99. Available at: http://link.springer.com/10.1245/s10434-015-5026-x.

4. Holter S, Borgida A, Dodd A, et al. Germline BRCA mutations in a large clinic-based cohort of patients with pancreatic adenocarcinoma. J. Clin. Oncol. 2015;33:3124–3129.

5. Salo-Mullen EE, O’Reilly EM, Kelsen DP, et al. Identification of germline genetic mutations in patients with pancreatic cancer. Cancer 2015;121:4382–8. Available at: http://doi.wiley.com/10.1002/cncr.29664.

6. Waddell N, Pajic M, Patch A, et al. Whole genomes redefine the mutational landscape of pancreatic cancer. Nature 2015;518:495–501. Available at: http://www.ncbi.nlm.nih.gov/pubmed/25719666.

7. Zhen DB, Rabe KG, Gallinger S, et al. BRCA1, BRCA2, PALB2, and CDKN2A mutations in familial pancreatic cancer: a PACGENE study. Genet. Med. 2014;17:569–77. Available at: http://www.ncbi.nlm.nih.gov/pubmed/25356972 [Accessed January 22, 2015].

8. Grant RC, Selander I, Connor AA, et al. Prevalence of Germline Mutations in Cancer Predisposition Genes in Patients With Pancreatic Cancer. Gastroenterology 2015;148:556–564. Available at: http://www.ncbi.nlm.nih.gov/pubmed/25479140 [Accessed January 22, 2015].

9. Roberts NJ, Norris AL, Petersen GM, et al. Whole genome sequencing defines the genetic heterogeneity of familial pancreatic cancer. Cancer Discov. 2015:166–176. Available at: http://cancerdiscovery.aacrjournals.org/cgi/doi/10.1158/2159-8290.CD-15-0402.
